# Bacteriocin-Producing Staphylococci and Mammaliicocci Strains for Agro-Food and Public Health Applications with Relevance of Micrococcin P1

**DOI:** 10.3390/antibiotics14010097

**Published:** 2025-01-16

**Authors:** Rosa Fernández-Fernández, Carmen Lozano, Allelen Campaña-Burguet, Carmen González-Azcona, Tamara Álvarez-Gómez, Rocío Fernández-Pérez, Raquel Peña, Myriam Zarazaga, Jaime Carrasco, Carmen Torres

**Affiliations:** 1 Area of Biochemistry and Molecular Biology, OneHealth-UR Research Group, University of La Rioja, 26006 Logroño, Spain; rosa.fernandez@unirioja.es (R.F.-F.); carmen.lozano@unirioja.es (C.L.); allelen.campana@unirioja.es (A.C.-B.); carmen.gonzalezaz@unirioja.es (C.G.-A.); tamara.alvarez@alum.unirioja.es (T.Á.-G.); myriam.zarazaga@unirioja.es (M.Z.); 2 Instituto de Ciencias de la Vid y del Vino (ICVV) (Universidad de La Rioja, Consejo Superior de Investigaciones Científicas (CSIC), Gobierno de La Rioja), 26007 Logroño, Spain; rocio.fernandez@unirioja.es; 3 Department of Microbiology and Parasitology, Instituto de Investigación Sanitaria de Navarra (IdiSNA), University of Navarra, 31008 Pamplona, Spain; rpenavillaf@alumni.unav.ed; 4 Department Ecology of Cultivated Mushrooms, Regional Institute for Agri-Food and Forest Research and Development (IRIAF), 16194 Cuenca, Spain; jccarrasco@jccm.es

**Keywords:** bacteriocin, staphylococcins, mammaliicoccins, bacteriocin-producing strains, antimicrobial activity, agro-food, public health

## Abstract

Antimicrobial-producing strains and their bacteriocins hold great promise for the control of bacterial diseases, being an attractive alternative to antibiotics. Thus, the aim of this study was to evaluate the inhibitory activity of 15 bacteriocin-producing staphylococci and mammaliicocci (BP-S/M) strains and their pre-purified extracts with butanol (BT) against a collection of 27 harmful or zoonotic strains (including Gram-positive/-negative bacteria and molds) with relevance in the public health and agro-food fields. These indicators (excluding Gram-negative strains) were grouped into seven categories based on their potential application areas: dairy livestock mastitis, avian pathogen zoonoses, swine zoonoses, food safety, aquaculture, wine making, and mushroom cultivation. In addition, cross-immunity assays between the BP-S/M strains were carried out to identify potential strain combinations to enhance their activity against pathogens. Finally, the hemolytic and gelatinase activities were tested in the BP-S/M strains. A strong inhibitory capacity of the BP-S/M strains was verified against relevant Gram-positive indicators, such as methicillin-resistant *Staphylococcus aureus*, *Listeria monocytogenes*, and *Clostridium perfringens*, among others, while no activity was detected against Gram-negative ones. Interestingly, several BT extracts inhibited the two mold indicators included in this study as representants of mushroom pathogens. The Micrococcin P1 producer *Staphylococcus hominis* C5835 (>60% of indicators were intensively inhibited by all the methods) can be proposed as a potential candidate for the control of bacterial diseases in the aforementioned categories alone or in combination with other BP-S/M strains (mainly with *Staphylococcus warneri* X2969). In this regard, five potential combinations of BP-S/M strains that enhanced their activity against specific pathogens were detected.

## 1. Introduction

The emergence of antibiotic resistance in commensal and pathogenic bacteria, due to the high use of antibiotics in humans and animals, has become an unresolved challenge to public health worldwide that needs to be addressed. In this respect, natural antimicrobial agents, including bacteriocins, have attracted extensive attention as a new microbial barrier in both the food and veterinary sectors [1]. Moreover, under the One Health approach, it is important to expand the study not only with human bacterial pathogens but also with those that cause severe diseases in animals and that affect the production of livestock industries (bovine, avian, or swine) with important economic activities worldwide [2,3].

Among the most relevant human opportunistic pathogens are *Escherichia coli* and *Staphylococcus aureus* [4], especially methicillin-resistant isolates (MRSA), which have been reported as an important target in both community and hospital settings, causing skin, wound, bloodstream, and other types of infections [5,6,7]. Moreover, these bacteria and other harmful microorganisms are also relevant in livestock production, highlighting *Streptococcus suis* in swine, *Clostridium perfringens* in poultry, and other Gram-positive/Gram-negative (G-positive/G-negative) pathogens engaged in bovine infections such as *S. aureus*, *Streptococcus uberis*, *Streptococcus dysgalactiae*, *Streptococcus agalactiae*, *E. coli*, *Klebsiella pneumoniae*, and *Mycoplasma* spp., among others. Concretely, *S. aureus* is responsible for about 50% of mastitis cases, and since this bacterium can inhabit the mammary gland and form abscesses, it is more difficult to combat upon using antibiotics [8,9]. In addition, *S. aureus* can also cause frequent problems in the food industry leading to important economic losses [10].

Potential alternatives to limit the use of antibiotics in the agro-food sector and in public health have been proposed, including antimicrobial peptides, bacteriophages, nanomedicines, probiotics, phytochemicals, and photodynamic light therapy, among others [11,12,13,14]. Bacteriocins are antimicrobial peptides produced by bacteria, mainly of ribosomal synthesis, with activity predominantly directed against bacteria closely related to the producing strain. There is a great diversity of bacteriocins, which can typically be classified into three classes based on their size, microbial target, mode of action, release, and immunity mechanisms. In addition, bacteriocins have different modes of action, the formation of pores in the target cell membrane being one of the most frequent. Commonly, bacteriocins are classified as Class I, post-transcriptionally modified and very diverse (lanthipeptides, head-to-tail cyclized peptides, and thiopeptides, among others); Class II, small and heat-stable; or Class III, large and thermally unstable peptides [1,15,16,17,18]. Moreover, these antimicrobial peptides are a viable strategy to replace conventional antibiotics or to potentiate their effects against pathogens, holding great promise (both purified and partially purified) in the control of harmful target bacteria.

Some *Staphylococcus* species, especially non-*aureus* staphylococci, are frequently found as commensals of humans and animals and are uncommonly associated with infections. Very recently, several coagulase-negative staphylococcal species have been included in a new genus, named *Mammaliicoccus*, which gathers, among others, the species *M. sciuri* (formerly *Staphylococcus sciuri*) [19]. The possible interest in bacteriocin-producing (BP) staphylococci and mammaliicocci (S/M) isolates or their antimicrobial compounds as antimicrobial, antitubercular, antifungal, antiviral, and/or anticancer strategies has been recently reported [20,21,22,23,24,25].

In this respect, a large collection of coagulase-positive and -negative staphylococcal strains (which include species now considered as mammaliicocci) was evaluated to detect and characterize commensal strains with antimicrobial activity. Based on the good results obtained in previous studies in terms of activity [26], added to the detection of bacteriocins at the genetic and protein level [27,28], we decided to select a collection of 15 BP-S/M strains with activity against G-positive bacteria (including different species of *Enterococcus*, *Staphylococcus*, and *Listeria*). Thus, in the present study, we aimed to further analyze the inhibitory capacities of the 15 selected BP-S/M strains (initially all of them identified as staphylococci) and their pre-purified bacteriocins against a wide collection of microorganisms used as indicators for agro-food and public health interest.

## 2. Results

### 2.1. Inhibitory Capacity of the BP-S/M Strains

Regarding the antimicrobial and antifungal activities of the 15 BP-S/M strains, Table 1 shows that all the 22 G-positive bacteria and the two molds were inhibited by at least one of the BP-S/M strains in one of the three conditions evaluated. Nevertheless, none of the BP-S/M strains showed antimicrobial activity (AA) against the three G-negative bacteria used as indicators in any of the assays evaluated. An example of the antimicrobial activity detected in this study by the three methods [*spot-on-lawn*, cell-free supernatants (CFS), and butanol (BT) extracts] is illustrated in Supplementary Figure S1.

#### 2.1.1. Antimicrobial Activity of BP-S/M Strains Against the Indicators

The BP-S/M strains showed interesting antimicrobial activities against the 27 indicator microorganisms. The percentages of the BP-S/M strains (out of the 15 tested) that showed AA against each indicator microorganism are presented in Table 1, differentiated by the three different methods and the main application fields. Those indicators that were inhibited by ≥60% of the BP-S/M strains are colored in dark grey. Regarding the *spot-on-lawn* method, seven indicator strains were the most inhibited: methicillin-resistant *S. aureus* (MRSA) C5313, C1570 and C1532 strains; *Listeria monocytogenes* CECT911; *Lactococcus garviae* Om-Pe-HK-61; *Pediococcus acidilactici* A101; and *Leuconostoc mesenteroides* A103. Considering the activity of CFS, the inhibition rates of each indicator, when positive, ranged from 27% to 47% (with the highest rate observed in the case of *L. monocytogenes*). Moreover, none of the CFS of the 15 BP-S/M strains exhibited activity against the *C. perfringens* X9740, *L. mesenteroides* A103, or MRSA C5313 and C1532 strains. Finally, the inhibitory capacity of the BT extracts against the G-positive strains included in this study was notably higher than that observed with the other methods. It is worth noting the high percentages of inhibition (up to 93%) detected in the BT extracts against some indicator bacteria classified into the dairy livestock mastitis, swine zoonoses, and food safety fields of application. Nevertheless, both the CFS and BT extracts revealed limited inhibitory capacities (percentages from 13% to 33%) against lactic acid bacteria included in the aquaculture and wine-making fields. Finally, the antifungal activities of the BT extracts against pathogens involved in mushroom cultivation while preventing both the spore germination and mycelium growth of *Trichoderma atroviride* TAV1 (27% of BP staphylococci) and *Cladobotryum mycophilum* CM13900 (40%) are highlighted (Table 1).

#### 2.1.2. Spectrum of Antimicrobial Activity of BP-S/M Strains Against Indicators

**Figure 1:**
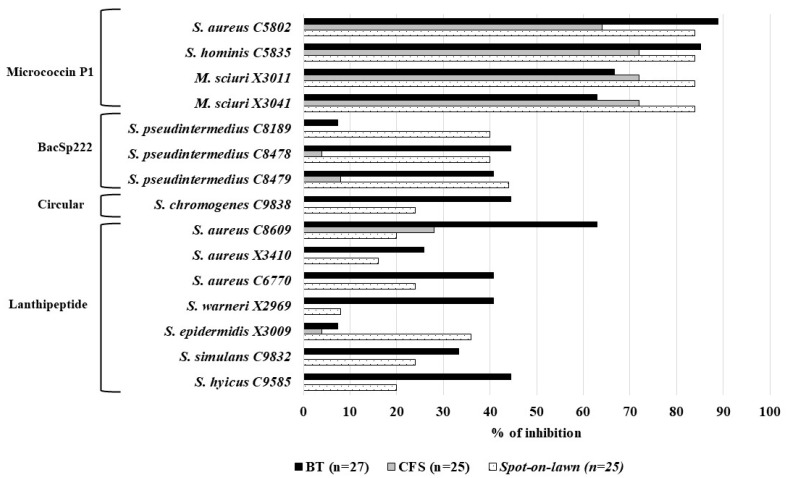
Spectrum of antimicrobial activity of the 15 bacteriocin-producing (BP) staphylococci and mammaliicocci (S/M) strains against the microorganisms used as indicators (number) considering three methods: *spot-on-lawn* (*n* = 25), cell-free supernatant (CFS) (*n* = 25), and butanol extraction (BT) (*n* = 27). The 15 BP-S/M strains were grouped according to the type of bacteriocin produced. The percentage of inhibition represents the percentage of the indicator microorganisms inhibited by each of the 15 BP-S/M strains. The antifungal activity of the 15 BP-S/M strains against the two molds included in this study was only evaluated with the BT extracts by disc diffusion assay.

The spectrum of the antimicrobial activity of the 15 BP-S/M strains (grouped by the type of bacteriocin they produce) against the microorganisms used as indicators considering three methods: *spot-on-lawn* (*n* = 25), cell-free supernatant (CFS) (*n* = 25), and butanol extraction (BT) (*n* = 27), is shown in Figure 1. High percentages of indicator microorganisms were inhibited by the BP-S/M strains when the *spot-on-lawn* method (up to 84%), BT extraction (up to 89%), and CFS extracts (until 72%) were used. Among all of them, the MP1 producers showed the widest spectrum of activity by the three methods considered in this study. In relation to the BacSp222 producers, two profiles of antimicrobial activity were observed (Figure 1): (i) the *S. pseudintermedius* C8478 and C8479 strains revealed very similar antimicrobial activity patterns by the three methods tested; (ii) *S. pseudintermedius* C8189 showed lower activity with the BT or CFS extracts than that of the other BacSp222 producers. The *S. chromogenes* C9838 strain, carrying a gene of a circular bacteriocin, showed a higher percentage of inhibition against the indicators using the BT extract in comparison with that of the *spot-on-lawn* assay. Finally, the lanthipeptide-producing strains revealed different patterns of activities by the *spot-on-lawn* assays or by testing their BT extracts (Figure 1).

Supplementary Figure S2 represents the spectrum of the antimicrobial activity of the 15 BP-S/M strains (A: *spot-on-lawn*) and their bacteriocins (B: BT extracts) against the indicator microorganisms grouped by the fields of potential application. It is interesting to highlight that the Micrococcin P1 (MP1)-producing S/M strains were active against almost all the indicator categories. Moreover, the *spot-on-lawn* assays clearly showed more activity against the avian-related indicators than the BT extracts. Conversely, the BT extracts seemed to have more activity against the swine and food-related indicators (Supplementary Figure S2).

### 2.2. Intensity of the Inhibitory Action of the BP-S/M Strains or Their Bacteriocin Extracts Against the Indicator Microorganisms

**Figure 2:**
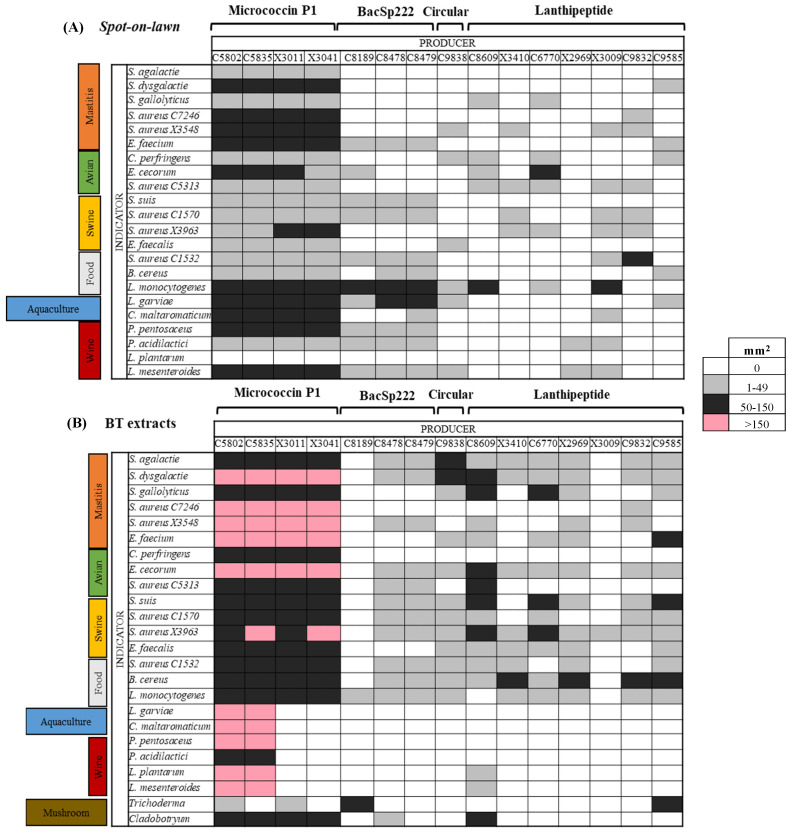
Intensity of inhibition of the 15 BP staphylococci and mammaliicocci (S/M) strains against the indicator microorganisms classified into seven categories based on their potential applications and represented in colors: two methods were used (numbers of indicators): (**A**) *spot-on-lawn* (*n* = 22), (**B**) BT extracts (*n* = 24). The intensity of the inhibitory activity was calculated by the inhibition zones as indicated in the Materials and Methods (in mm^2^): white (0); light grey (1–49); dark grey (50–150); rose (>150).

The intensity of inhibition was calculated considering the inhibitory haloes of the 15 BP-S/M strains (*spot-on-lawn*) or their pre-purified extracts (BT) obtained against the indicator strains grouped into seven categories (related to the main fields of interest for potential application) and is represented in Figure 2. In this respect, the BT extracts showed special activity (with high intensity) against the indicators of interest for dairy livestock mastitis, aquaculture, and wine making (Figure 2B). Interestingly, the BT extracts of eight BP-S/M strains were active against one or two major mushroom pathogens, and, concretely, the MP1 producers (C5802, C5835, X3011, X3041) and lanthipeptide-like producers (C8609 and C9585) inhibited the growth in the cohabitant-symbiotic microbiota of the *Cladobotryum* and *Trichoderma* mycelia, respectively.

Moreover, the activity of the MP1 producers should be highlighted, which inhibited at least one indicator of each of the categories evaluated and revealed the strongest antimicrobial activity both by *spot-on-lawn* assays and BT extracts (Figure 2), especially the *S. aureus* C5802 and *S. hominis* C5835 strains. Conversely, the *S. pseudintermedius* C8189, *S. aureus* X3410, and *S. epidermidis* X3009 strains showed lower inhibitory capacities, acting only against one to four indicator categories, and also with reduced halo sizes.

### 2.3. Cross-Immunity and Potential Combinations of BP Strains

**Figure 3:**
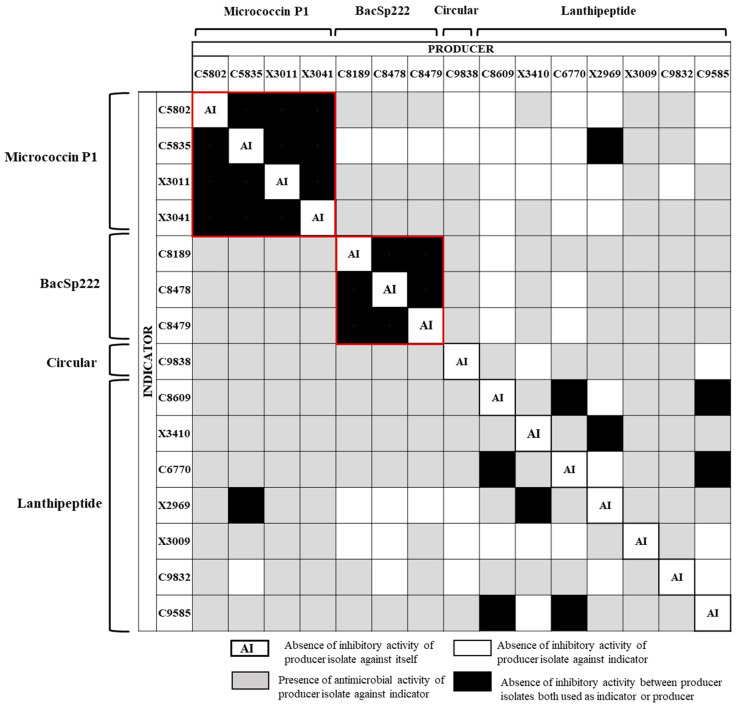
Cross-immunity assay between the 15 bacteriocin producing (BP) staphylococci/mammaliicocci (S/M) strains classified according to the bacteriocins produced. All BP-S/M strains were used both as producers (in columns) and as indicators (in lines) in this assay. The BP-S/M strains that lacked antimicrobial activity when they were used both as indicator or producer bacteria are marked with a red box, for cases in which they carried exactly the same bacteriocin (MP1 and BacSp222).

In order to evaluate the relatedness between the bacteriocins of the 15 BP-S/M strains, a cross-immunity assay was performed using all these BP strains both as bacteriocin producers and indicator bacteria (Figure 3). The white box indicates the absence of inhibitory activity in the pair producer–indicator bacteria, the grey box represents the existence of antimicrobial activity, and the black box indicates the absence of inhibitory activity between two BP-S/M strains both used as producers and indicator bacteria. As expected, the MP1 and BacSp222 producers did not exhibit inhibitory activity against strains of the same BP group due to the production of identical bacteriocins (boxes marked in red). The four MP1-producing strains inhibited almost all the BP-S/M strains (except other MP1 producers). Interestingly, all the MP1-producing strains were immune to the inhibitory activity of C8609, C6770, and X2969 as BP strains (carriers of genes encoding lanthipeptides) (Figure 3). Noteworthy, the MP1-producing *S. hominis* C5835 was immune to the inhibitory activity of 13 (including itself) of the BP-S/M strains (except for X3009 and C9832, carriers of genes of lanthipeptides), and what is more, it did not show inhibitory activity when testing against *S. warneri* X2969. Moreover, the three *S. pseudintermedius* producers of the BacSp222 bacteriocin were immune to the inhibitory activity of *S. aureus* C8609 (lanthipeptide-like-producer strain).

In this respect, considering the mutual interactions among the BP-S/M strains, the following potential BP-S/M combinations can be proposed: (i) two lanthipeptide-producing bacteria, such as C6770 + C8609, C6770 + C9585, C8609 + C9585, or X3410 + X2969; (ii) the lanthipeptide-producing *S. warneri* X2969 with the MP1-producing *S. hominis* C5835. In all these combinations, the two BP-S/M strains did not show inhibitory activity against the partner (marked in the black box in Figure 3).

### 2.4. Preliminary Characterization of Pathogenesis

A total of six out of the 15 BP-S/M strains were β-hemolytic, and all were coagulase-positive: three *S. aureus* (C8609, C6770, C5802) and three *S. pseudintermedius* (C8189, C8478, C8479). All the BP-producing coagulase-negative were negative for β-hemolysis. Moreover, five strains revealed gelatinase activity, including both coagulase-positive (three *S. pseudintermedius*: C8189, C8478, C8479) and coagulase-negative strains (*S. hyicus* C8581 and *S. chromogenes* C9838). Therefore, the subsequent screening of the safety and security of BP-S/M strains should be performed in the future to ensure their possible uses as protective cultures.

## 3. Discussion

Currently, the use of antimicrobial products with an environmental and respectful background is demanded. Bacteriocins have enormous research and potential application and hold great promise for the control of bacterial diseases, being an attractive alternative to antibiotics or chemical preservatives, and can also be used as semi-purified or bioactive compounds [29]. Several studies have indicated the health and economic benefits of bacteriocins or BP-S/M strains as protective cultures and as preventive treatments against diseases or in combination with other antimicrobial compounds for livestock production [1,30,31,32,33,34,35,36,37,38]. However, their application is still limited, at least regarding staphylococci and mammaliicocci.

The discovery and characterization of new antimicrobials, such as S/M bacteriocins, will likely become an important step in the fight against antimicrobial resistance. In this respect, the present study aims to contribute to the knowledge of the potential applications of staphylococcins (including those of mammaliicocci; in this case, we propose to use the term mammaliicoccin) and the BP-S/M strains. This study shows the interesting antimicrobial activities of BP-S/M strains verified by several methods against harmful microorganisms, including MDR bacteria, involved in relevant infections and important problems of agro-food application fields such as dairy livestock mastitis, poultry pathogen zoonoses, swine zoonoses, food safety, aquaculture, mushroom cultivation, and wine making. Overall, each G-positive bacteria tested as an indicator was inhibited by the BT extract of at least two BP-S/M strains, whereas none of the producers revealed antimicrobial activity against the G-negative indicators. These results are consistent with the available literature, wherein it is assumed that commensal bacteriocin-producing strains inhibit specific colonizing pathogens [39]. Concretely, staphylococcins often show antimicrobial activity against related bacteria, and normally G-positive bacteria [40].

As expected, wide inhibitory activities were detected when BT extracts were used. It is worth noting that 10 BP-S/M strains had antimicrobial activity against more than 40% of the indicators, regardless of their assigned category (*S. aureus* C5802; *S. hominis* C5835; *M. sciuri* X3041 and X3011; *S. pseudintermedius* C8478 and C8479; *S. aureus* C8609 and C6770; *S. warneri* X3009; and *S. hyicus* C9585). These BP strains produced Micrococcin P1, BacSp222, and lanthipeptide-like bacteriocins, which have previously been reported to inhibit critical G-positive harmful bacteria such as *Mycobacterium tuberculosis*, MRSA, *Listeria monocytogenes*, vancomycin-resistant enterococci, and *Clostridium difficile*, among others [16,41,42,43].

Taking into account the results obtained in relation to the harmful bacteria of interest in livestock production, we highlight that all the BP-S/M strains evaluated in this study inhibited at least one indicator strain included in the bovine mastitis and swine zoonoses categories, both considered of great relevance in livestock production. Moreover, the assays performed in this study revealed that the strains included in the swine pathogen category were the most susceptible to BT extracts, while both BP-S/M strains (*spot-on-lawn*) and their bacteriocins (BT extracts) could be active against strains included in the dairy mastitis pathogen cathegory. In this respect, the use of bacteriocins as natural antimicrobial substances for mastitis control and prevention has already been considered [38,44,45]. A recent ex vivo study proposes the use of staphylococcins against pathogens isolated from bovine mastitis, evidencing that, after 24 h of incubation, aureocin A53 caused a strong reduction in staphylococcal or streptococcal populations, eliminating all the detectable viable cells [46]. In addition, it is of great interest to solve the problem of the community transmission of *S. aureus*, and especially MRSA-CC398, between pigs and humans, a livestock-associated lineage considered an emerging public health problem. On this basis, a recent ex vivo and in vivo murine model study confirmed the protective role of the porcine skin microbiota, including bacteria-producing antimicrobial substances, against MRSA in a colonization model [47]. Here, cross-immunity assays allowed us to detect five bacterial combinations of lanthipeptide-producing strains and the MP1-producing *S. hominis* C5835 strain of interest for future studies by enhancing their antimicrobial activity against MRSA, among other pathogens. Moreover, in a previous study [28], the MP1-producing C5835 strain and its pre-purified extract revealed an intense inhibitory capacity against an MRSA-MDR-CC398 strain, which allows us to consider this producing strain as a good candidate for potential applications. Similar results have recently been presented, suggesting the use of BP strains with synergistic interactions in vivo as consortium probiotic supplements to promote the health of piglets [48].

Regarding poultry, it is important to highlight the potential use of BP strains as suitable alternatives to antibiotics to combat or prevent infections of relevant pathogens in poultry farming, such as *C. perfringens* and MRSA, as well as for improving the performance and productivity of birds [49,50]. In this respect, the results presented in this study revealed BP-S/M strains with potent inhibitory activity by different methods against indicators included in the avian pathogen zoonoses category, such as *C. perfringens*, MRSA, and *E. cecorum*. Concretely, it is worth noting that the four MP1-producing strains (C5802, C5835, X3041, and X3011) and three lanthipeptide-like-producing (C8609, C6770, and X3410) S/M strains inhibited all three indicators of the avian pathogen category tested by the *spot-on-lawn* assay.

The aquaculture system field was also considered in this study and one *Lactococcus garviae* strain was included as an indicator, as it is regarded as an emerging zoonotic pathogen [51]. Focusing on our results, the *Lactococcus garviae* strain was inhibited by 60% of the BP staphylococci (*spot-on-lawn*), as well as by their extracts. The potential use of BP-S/M strains and their bacteriocins in this sector could therefore be proposed for further studies.

Regarding the food industry, foodborne disease causes an estimated 48 million illnesses and 3000 deaths annually, with important economic costs [52]. Among the most harmful microorganisms, the negative effects of *Salmonella*, *L. monocytogenes*, *S. aureus*, *C. perfringens*, and *E. coli* contamination can be highlighted [10]. In this study, several extracts demonstrated interesting inhibitory effects against the G-positive strains included in the food pathogen category. Attending to the food industry, nisin is undoubtedly the most widely used and effective bacteriocin against foodborne pathogens, although the use of other bacteriocins has gained importance due to their wide range of applications in the food industry [53]. In this respect, a previous study carried out by our group compared the antimicrobial activity of non-purified extracts of MP1-producing strains with that of a non-pure nisin extract (2.5%, equivalent to 5 µg/mL) and the relevant antimicrobial activities of MP1 were detected against *S. aureus* MRSA and *L. monocytogenes* [28].

Moreover, lactic acid bacteria (LAB) have been widely used in the food industry and concretely represent a key factor for wine production responsible for malolactic fermentation [54]. In this respect, synergism between commercial bacteriocins and preservatives such as sulfur dioxide has been reported in controlling the growth of LAB in the different steps of wine production: *Oenococcus*, *Lactobacillus*, *Leuconostoc*, and *Pediococcus* [55]. In this study, we detected BP-S/M strains and some of their BT extracts with interesting activities against LAB included in the wine-making category, revealing their potential interest in this field of application.

With respect to mushroom cultivation, fungal diseases are among the most serious disorders damaging and affecting the quality of the yields of relevant crops such as mushroom [56,57]. However, the scarce range of available products in addition to the occurrence of resistance derived from the continuous exposure to the same active substances evidence the need for alternative treatments [58]. Concretely, we focus on two of the most harmful fungal diseases affecting cultivated mushrooms: cobweb disease (*Cladobotryum* spp.) and green mold disease (*Thrichoderma* spp.). Interestingly, antifungal activity was observed in six out the 15 BP-S/M strains tested in this study towards these mycoparasites, also inhibiting the mycelial growth of *Cladobotryum mycophilum* by the noted antagonism towards its respective cohabitant-symbiotic microbiota, suggesting that the growth of this harmful fungi could be repressed by the observed detrimental effect on its mutualistic microbiota. Thus, bacteriocins could be of interest as a biological control agent of some mycoparasites by means of their detrimental action on essential bacteria conforming to the pathogen microbiome. Nevertheless, more research should be performed in this field to deepen the understanding of these activities and the potential mechanisms of action. This is a singular observation in our approach since, commonly, mycoparasites are commercially treated by chemical fungicides, but the scarcely known fungal–bacterial interactions could play a role in facilitating pathogen control through active bacteriocin producers. This configuration may have a direct impact on the yield performance and disease occurrence [59,60], contributing to enhancing the production of protein alternatives to meat-based, Western diets.

Overall, the present study demonstrates that the use of both BP-S/M strains and their bacteriocins as pre-purified BT extracts could be considered in further applications. Specifically, the wide spectrum and intense inhibitory activity of the MP1 producers against all the indicator categories and by all the conditions evaluated are remarkable.

Micrococcin P1 is a 26-membered ring thiopeptide isolated from a wide variety of sources ranging from soil to cheese. MP1 has been reported to primarily inhibit G-positive bacteria, although it has also been described as a good antitubercular compound [43]. Regarding its mode of action, MP1 is able to inhibit bacterial protein synthesis, especially the elongation step, by binding to the GTPase-associated region of the ribosome involving the protein L11 and related rRNA complex [43,61]. Additionally, it has been reported that mutations in ribosomal protein L11 in mycobacteria is associated with resistance to MP1 [43].

Finally, it is important to consider the limitations of this study regarding the limited number of indicators in each category and the criteria used to establish the respective groups. Certainly, the inhibitory activities observed in this study depend on the established categories, on the producing strains, and on the type of bacteriocin; however, the results elucidate promising alternatives to antibiotics in the agro-food and public health sectors.

## 4. Materials and Methods

### 4.1. Bacteriocin-Producing and Indicator Strains

The 15 BP-S/M strains included in this work were obtained and well characterized as bacteriocin producers at the genetic and protein level in previous studies [26,27,28]. A summary of their molecular characterization after whole-genome analysis and their contents of bacteriocins is shown in Supplementary Table S1, and a briefly summarization is shown in Table 2. These BP-S/M strains contained the bacteriocin gene clusters encoding the MP1 thiopeptide (*S. aureus* C5802, *Staphylococcus hominis* C5835, and *Mammaliicoccus sciuri* X3041 and X3011); lanthipeptides (*S. aureus* C8609, *S. aureus* X3410, *S. aureus* C6770, *Staphylococcus epidermidis* X3009, *Staphylococcus warneri* X2969, *Staphylococcus simulans* C9832, and *Staphylococcus hyicus* C9585); circular bacteriocins (*Staphylococcus chromogenes* C9838); and the BacSp222 (*S. pseudintermedius* C8189, C8478, and C8479).

The inhibitory activity of these 15 strains was evaluated against selected harmful strains used as indicators (22 G-positive strains, three G-negative strains, and two molds), including multidrug-resistant (MDR) and zoonotic strains of relevance. Indicator strains were selected based on their species and/or antimicrobial resistance phenotypes and were classified into seven categories based on their main fields of potential application (Table 1): dairy livestock mastitis, avian pathogen zoonoses, swine zoonoses, food safety, aquaculture, wine making, and mushroom cultivation. Although, for simplicity, each of these indicator strains was categorized into only one potential application field, it is important to remark that they could be included for the study of several. In addition to most of the indicator microorganisms were G-positive bacteria, and three G-negative bacteria (*Escherichia coli*, *Salmonella*, and *Pseudomonas aeruginosa*) were also included as indicators and were considered separately (outside the potential application fields). The antimicrobial activity of these BP-S/M strains against six indicator bacteria of the genera *Staphylococcus*, *Enterococcus*, and *Listeria* has already been tested in previous studies [28].

Moreover, *Cladobotryum mycophilum* CM13900 and *Trichoderma atroviride* TAV1, isolated from commercial mushroom farms in La Rioja (Spain), were used as indicators to study the possible antifungal activity (AF) of the BP-S/M strains. These mycopathogens are responsible for cobweb and green mold on mushroom farms.

### 4.2. Culture Media and Growth Conditions

Brain Heart Infusion (BHI) (Condalab, Madrid, Spain) was used as the standard growth medium for both liquid and solid cultures (1.5% of agar). Antimicrobial activity determinations were performed by the “*spot-on-lawn*” assay in plates containing one layer of 10 mL of tryptic soy agar (TSA) supplemented with 0.3% of yeast extract (Condalab Spain) and a second layer of 5 mL of tryptic soy broth (TSB) supplemented with 0.3% yeast extract (Condalab, Madrid, Spain) and 0.7% agar tempered at 45 °C.

Anaerobic conditions were used for the *Clostridium perfringens*, *Pediococcus* spp., *Lactobacillus plantarum*, and *Leuconostoc mesenteroides* indicators. Columbia agar with 5% sheep blood (BioMérieux, France) was used when the indicator strain was *Streptococcus suis* and potato dextrose agar media (PDA) was used in the case of molds. The 15 BP-S/M strains and the indicator microorganisms were grown in BHI agar incubated overnight at 37 °C during 24 h, except those indicators selected as representatives for mushroom cultivation and aquaculture applications, which were incubated at 25 °C during 24 h to 96 h in the case of molds and 48 h for lactic acid bacteria.

### 4.3. Detection of Antimicrobial Activity (AA)

The AA of the BP-S/M strains was evaluated by the *spot-on-lawn* method (*n* = 25 indicators), and the inhibitory capacity of two different bacterial extracts: CFS (*n* = 25) and BT (*n* = 27 indicators) was tested by agar diffusion assays. The two molds were only tested as indicators with the BT extracts.

For the *spot-on-lawn* method, indicator strains were resuspended in Brain Heart Infusion (BHI) broth to obtain a 0.5 McFarland (10^8^ CFU). Later, 10 μL of these cultures were added to tubes containing 5 mL of semisolid TSA (tryptic soy broth (TSB) containing 0.5% yeast extract and 0.7% agar), and the mixtures were poured onto TSA agar plates with 0.3% yeast extract. After that, the 15 BP-S/M strains were spotted on the surface of each plate (previously seeded with each indicator strain) using sterile toothpicks, and plates were incubated overnight at 37 °C and analyzed for AA. All determinations were performed in duplicate.

Regarding agar diffusion assays, CFS and BT extracts were obtained from the 15 BP-S/M strains. For preparation of CFS, strains were grown in BHI broth medium for 24 h at 37 °C under continuous shaking, centrifuged (4500 rpm, 10 min), and sterilized by filtration through a low-protein-binding 0.45 µm Millipore filter. BT extracts were obtained by incubating boiling sterilized CFS (100 °C, 5 min) with 1-butanol at a ratio of 1:3 and were shacked for 1 h at 37 °C. Samples were centrifuged at 4500 rpm for 15 min, the aqueous phase was discarded after centrifugation, and the organic phase was evaporated at 44 °C under reduced pressure. The BT extracts were resuspended in the minimum needed volume of dimethyl sulfoxide (DMSO) (0.7–0.9 mL). Indicator strains were resuspended in 0.9% sterile saline solution at a bacterial concentration of 0.5 McFarland turbidity and were seeded on TSA plates. Wells of 6 mm diameter were punched into the inoculated agar plates and filled with 50 µL of the CFS samples, whereas 10 µL of the BT extract was added to a blank disc. Plates were incubated overnight at the corresponding temperatures, and diameters of inhibition zones were measured (mm) and recorded. All determinations were repeated.

### 4.4. Detection of Antifungal Activity (AF)

The mold spores were collected in sterile water and rubbed onto the PDA surface with a glass rod (100 µL per plate). The spore suspensions were quantified by using a counting chamber, adjusted to 10^4^ spores/mL, and used as inoculum. The disc diffusion assay was used to evaluate the AF. Thus, 10 µL of BT extracts of the 15 BP-S/M strains was added to sterile discs. Dry discs were placed in PDA plates inoculated with mold spore suspension.

### 4.5. Intensity of the Inhibitory Capacity

The inhibition intensity of the 15 BP-S/M strains against the 27 indicator strains was determined considering the inhibition diameter by measuring the haloes in millimeters (mm) after *spot-on-lawn* and BT assays, as follows: area of zone πr^2^−area of well πr^2^ [62].

### 4.6. Cross-Immunity Among the BP Staphylococci

The cross-immunity assay was performed to determine the relatedness of the bacteriocins produced in the selected collection of BP-S/M strains (although it belongs to the same class of antimicrobial peptides)**.** Moreover, the cross-immunity assay allows us to propose bacterial combinations to enhance their own activities against other bacteria. Thus, the 15 BP-S/M strains included in this study were evaluated to explore their antimicrobial activity by the *spot-on-lawn* method described above using each BP-S/M strain both as a producer and target indicator strain [62].

### 4.7. Hemolysis and Gelatinase Activities

Overnight cultures of BP-S/M strains in BHI broth were spotted onto Columbia agar with 5% sheep blood (BioMérieux, France) and then incubated at 37 °C overnight. Hemolytic activities of the strains were recorded by the presence of beta (β) hemolysis when detecting a clear and complete hemolysis of blood cells around the BP-inoculated strain.

Moreover, gelatinase activity was evaluated in BP-S/M strains, which were spotted onto TSA plates containing 1.5% of skim milk and incubated overnight at 37 °C. Then, plates were cooled to room temperature for 2 h and evaluated for the appearance of a transparent halo around the colonies (positive for gelatinase test) [49].

## 5. Conclusions

The present study shows the promising antimicrobial capacity of both BP-S/M strains and their bacteriocins (pre-purified BT extracts) against harmful microorganisms classified into different categories within the agro-food and public health fields of application. In addition, cross-immunity assays helped us to propose five potential combinations of BP-S/M strains to enhance their activity against specific pathogens. Finally, the BP-coagulase-negative and mammaliicocci strains lacked beta-hemolytic and gelatinase activities, so further studies should be performed to deepen the applicability of the BP-S/M strains and their bacteriocins as protective cultures and antimicrobial agents, respectively. In addition to all the above, the three coagulase-negative producers of the MP1 bacteriocin, and especially the *S. hominis* C5835 strain, and their staphylococcins have shown themselves to be good candidates for agro-food applications due to their wide spectrum and high intensity of inhibition, especially against indicators included in the dairy livestock mastitis, avian pathogen zoonosis, aquaculture, and wine-making categories, alone or in combination with other BP staphylococci (*S. warneri* X2969).

## Figures and Tables

**Table 1:** Percentages of the 15 bacteriocin-producing (BP) staphylococci and mammaliicocci (S/M) strains with inhibitory activity against each of the 27 indicator strains classified into eight categories, using three methods (*spot-on-lawn*; cell-free supernatant, CFS; butanol extracts, BT).

Main Field of Interest	Indicator Strains ^b^	Percentage of BP Strains with Inhibitory Activity Against the Indicator ^a^		
		*Spot-on-lawn*	CFS	BT
Dairy livestock mastitis	*Streptococcus agalactie* X9738	27	40	87
	*Streptococcus dysgalactie* X9739	33	27	87
	*Streptococcus gallolyticus* X9742	40	33	60
	*Staphylococcus aureus* C7246 MRSA ^b^	33	27	33
	*S. aureus* X3548 MRSA ^b^	53	27	60
	*Enterococcus faecium* C2321 VanR ^b^	53	27	60
Avian pathogen zoonoses	*Clostridium perfringens* X9740	53	0	27
	*Enterococcus cecorum* X3809	47	33	87
	*S. aureus* C5313 MRSA ^b^	60	0	47
Swine zoonoses	*Streptococcus suis* X2060	47	27	80
	*S. aureus* C1570 MRSA-CC398 ^b^	67	27	73
	*S. aureus* X3963 MRSA ^b^	53	33	93
	*Enterococcus faecalis* C9951 LZD-R ^b^	33	27	67
Food safety	*S. aureus* C1532 MRSA ^b^	60	0	73
	*Bacillus cereus* X10062	47	27	87
	*Listeria monocytogenes* CECT911	73	47	73
Aquaculture	*Lactococcus garviae* Om-Pe-HK-61	60	27	13
	*Carnobacterium maltaromaticum* St-PS-HK-63	40	27	13
Wine making	*Pediococcus pentosaceus* A100	47	27	13
	*Pediococcus acidilactici* A101	60	27	13
	*Lactobacillus plantarum* A102	0	33	20
	*Leuconostoc mesenteroides* A103	67	0	20
Mushroom cultivation	*Trichoderma atroviride* TAV1	NT	NT	27
	*Cladobotryum mycophilum* CM13900	NT	NT	40
Gram-negative ^c^	*Salmonella* spp. X10061	0	0	0
	*Escherichia coli* ATCC25922	0	0	0
	*Pseudomonas aeruginosa* PAO1	0	0	0

^a^ The indicator strains inhibited by ≥60% of the BP-S/M strains are marked with the dark-grey color, those inhibited by 20% to 60% of the BP-S/M strains are marked in the light-grey color, and those inhibited by less than 20% of the BP-S/M strains are marked in the white color. ^b^ For some indicator strains, specific characteristics are included: MRSA: methicillin-resistant *Staphylococcus aureus*; VanR: vancomycin-resistant; LZD-R: linezolid-resistant; CC: clonal complex. ^c^ Gram-negative bacteria were considered separately due to the fact they were not inhibited by any of the BP-S/M strains included in this study or by their pre-purified extracts. Abbreviations: CECT, Spanish Type Culture Collection; ATCC, American Type Culture Collection; NT, not tested.

**Table 2:** Collection of the 15 bacteriocin-producing (BP) strains included in this study ^a^.

ID Code	Specie	Origin	Bacteriocin Detected
C5802	*S. aureus*	Environment–water	Micrococcin P1
C5835	*S. hominis*	Environment–water	Micrococcin P1
X3011	*S. sciuri* ^b^	Food–chicken	Micrococcin P1
X3041	*S. sciuri* ^b^	Food–chicken	Micrococcin P1
C8189	*S. pseudintermedius*	Human–dog	BacSp222
C8478	*S. pseudintermedius*	Human–dog	BacSp222
C8479	*S. pseudintermedius*	Human–dog	BacSp222
C9838	*S. chromogenes*	Wild animal–mammal	Circular
C8609	*S. aureus*	Wild animal–mammal	Lanthipeptide
X3410	*S. aureus*	Food–chicken	Lanthipeptide
C6770	*S. aureus*	Wild animal–mammal	Lanthipeptide
X2969	*S. warneri*	Food–chicken	Lanthipeptide
X3009	*S. epidermidis*	Food–chicken	Lanthipeptide
C9832	*S. simulans*	Wild animal–mammal	Lanthipeptide
C9585	*S. hyicus*	Wild animal–mammal	Lanthipeptide

^a^ Data on the strains were obtained in previous studies [27,28]. ^b^ *S. sciuri* is now included in the new *Mammaliicoccus* genus and is considered *M. sciuri*.

## Data Availability

All data generated and reported during this study are included or indicated in the published article.

## Supplementary Materials

The following supporting information can be downloaded at https://www.mdpi.com/article/10.3390/antibiotics14010097/s1, Table S1: Characteristics of the 15 bacteriocin-producing (BP) staphylococci and mammaliicocci (S/M) strains included in this study. Figure S1: Antimicrobial activity of five bacteriocin-producing staphylococci and mammaliicocci strains against different indicator bacteria represented from left to right (1–3): 1. bacteria (*spot-on-lawn*), 2. cell-free supernatant (diffusion assay), and 3. butanol extracts. (A) *Staphylococcus aureus* C5802 against MRSA X3963; (B) *Staphylococcus hominis* C5835 against *Enterococcus faecalis* C9951; (C) *Mammaliicoccus sciuri* X3011 against *Listeria monocytogenes*; (D) *Staphylococcus aureus* C8609 against *Streptococcus gallolyticus*; (E) *Mammaliicoccus sciuri* X3041 against *Bacillus cereus.* Figure S2: Spectrum of antimicrobial activity of the 15 bacteriocin-producing (BP) staphylococci/mammaliicocci (S/M) strains against the collection of microorganisms used as indicators considering the seven categories of application for which they were selected (marked in different colors). The 15 BP-S/M strains were grouped according to the type of bacteriocin produced. The figure represents the percentage of indicator strains inhibited by each of the BP staphylococci organized by the two methods (number of indicators tested): (A): *spot-on-lawn* (*n* = 22); (B): butanol extracts (BT, *n* = 24).

## References

[1] Soltani S., Biron E., Ben Said L., Subirade M., Fliss I. (2022). Bacteriocin-Based Synergetic Consortia: A Promising Strategy to Enhance Antimicrobial Activity and Broaden the Spectrum of Inhibition. Microbiol. Spectr..

[2] Fairbrother J.M., Nadeau É., Gyles C.L. (2005). *Escherichia coli* in postweaning diarrhea in pigs: An update on bacterial types, pathogenesis, and prevention strategies. Anim. Health Res. Rev..

[3] Gottschalk M., Segura M., Zimmerman J.J., Karriker L.A., Ramirez A., Schwartz K.J., Stevenson G.W., Zhang J. (2019). Streptococcosis. Diseases of Swine.

[4] Lagha B.A., Haas B., Gottschalk M., Grenier D. (2017). Antimicrobial potential of bacteriocins in poultry and swine production. Vet. Res..

[5] World Health Organization (WHO) (2014). Antimicrobial Resistance: Global Report on Surveillance.

[6] ECDC (European Centre for Disease Prevention and Control), EFSA (European Food Safety Authority), EMA (European Medicines Agency) Joint Scientific Report of ECDC, EFSA and EMEA on Methicillin Resistant *Staphylococcus aureus* (MRSA) in Livestock, Companion Animals and Foods. 2009, EFSA-Q-2009-00612 (EFSA Scientific Report) and EMEA/CVMP/SAGAM/62464/2009. https://www.efsa.europa.eu/en/efsajournal/pub/rn-301.

[7] European Centre for Disease Prevention and Control (ECDC) (2015). Antimicrobial Resistance Surveillance in Europe 2014.

[8] Gomes F., Henriques M. (2016). Control of Bovine Mastitis: Old and Recent Therapeutic Approaches. Curr. Microbiol..

[9] Mella A., Ulloa F., Valdés I., Olivares N., Ceballos A., Kruze J. (2017). Evaluation of a new vaccine against *Staphylococcus aureus* mastitis in dairy herds of southern Chile. I. Challenge trial. Austral J. Vet. Sci..

[10] Gallo M., Ferrara L., Calogero A., Montesano D., Naviglio D. (2020). Relationships between food and diseases: What to know to ensure food safety. Food Res. Int..

[11] Wang C.H., Hsieh Y.H., Powers Z.M., Kao C.Y. (2020). Defeating Antibiotic-Resistant Bacteria: Exploring Alternative Therapies for a Post-Antibiotic Era. Int. J. Mol. Sci..

[12] Anyaegbunam N.J., Anekpo C.C., Anyaegbunam Z.K.G., Doowuese Y., Chinaka C.B., Odo O.J., Sharndama H.C., Okeke O.P., Mba I.E. (2022). The resurgence of phage-based therapy in the era of increasing antibiotic resistance: From research progress to challenges and prospects. Microbiol. Res..

[13] Mba I.E., Nweze E.I. (2022). Antimicrobial Peptides Therapy: An Emerging Alternative for Treating Drug-Resistant Bacteria. Yale J. Biol. Med..

[14] Mba I.E., Nweze E.I., Hameed S., Rehman S. (2022). Application of Nanotechnology in the Treatment of Infectious Diseases: An Overview. Nanotechnology for Infectious Diseases.

[15] Darbandi A., Asadi A., Mahdizade A.M., Ohadi E., Talebi M., Halaj Z.M., Darb E.A., Ghanavati R., Kakanj M. (2022). Bacteriocins: Properties and potential use as antimicrobials. Clin. Lab. Anal..

[16] Huang F., Teng K., Liu Y., Cao Y., Wang T., Ma C., Zhang J., Zhong J. (2021). Bacteriocins: Potential for Human Health. Oxidative Med. Cell. Longev..

[17] Telhig S., Ben Said L., Torres C., Rebuffat S., Zirah S., Fliss I. (2022). Evaluating the Potential and Synergetic Effects of Microcins against Multidrug-Resistant *Enterobacteriaceae*. Microbiol. Spectr..

[18] Gillor O., Ghazaryan L. (2007). Recent Advances in Bacteriocin Application as Antimicrobials. Recent Pat. Anti-Infect. Drug Discov..

[19] Madhaiyan M., Wirth J.S., Saravanan V.S. (2020). Phylogenomic analyses of the *Staphylococcaceae* family suggest the reclassification of five species within the genus *Staphylococcus* as heterotypic synonyms, the promotion of five subspecies to novel species, the taxonomic reassignment of five *Staphylococcus* species to *Mammaliicoccus* gen. nov.; and the formal assignment of *Nosocomiicoccus* to the family *Staphylococcaceae*. Int. J. Syst. Evol. Microbiol..

[20] Dekham K., Jones S.M., Jitrakorn S., Charoonnart P., Thadtapong N., Intuy R., Dubbs P., Siripattanapipong S., Saksmerprome V., Chaturongakul S. (2023). Functional and genomic characterization of a novel probiotic *Lactobacillus johnsonii* KD1 against shrimp WSSV infection. Sci. Rep..

[21] Khusro A., Aarti C., Dusthackeer A., Agastian P. (2018). Anti-tubercular and probiotic properties of coagulase-negative staphylococci isolated from Koozh, a traditional fermented food of South India. Microb. Pathog..

[22] Khusro A., Aarti C., Mahizhaveni B., Dusthackeer A., Agastian P., Esmail G.A., Ghilan A.K.M., Al-Dhabi N.A., Arasu M.V. (2020). Purification and characterization of anti-tubercular and anticancer protein from *Staphylococcus hominis* strain MANF2: In silico structural and functional insight of peptide. Saudi J. Biol. Sci..

[23] Cebrián E., Núñez F., Gálvez F.J., Delgado J., Bermúdez E., Rodríguez M. (2020). Selection and Evaluation of *Staphylococcus xylosus* as a Biocontrol Agent against Toxigenic Moulds in a Dry-Cured Ham Model System. Microorganisms.

[24] Mangrolia U., Osborne W.J. (2020). *Staphylococcus xylosus* VITURAJ10: Pyrrolo [1,2α] pyrazine-1,4-dione, hexahydro-3-(2-methylpropyl) (PPDHMP) producing, potential probiotic strain with antibacterial and anticancer activity. Microb. Pathog..

[25] Fernández-Fernández R., Lozano C., Reuben R.C., Ruiz-Ripa L., Zarazaga M., Torres C. (2023). Comprehensive Approaches for the Search and Characterization of Staphylococcins. Microorganisms.

[26] Fernández-Fernández R., Lozano C., Eguizábal P., Ruiz-Ripa L., Martínez-Álvarez S., Abdullahi I.N., Zarazaga M., Torres C. (2022). Bacteriocin-Like Inhibitory Substances in Staphylococci of Different Origins and Species With Activity Against Relevant Pathogens. Front. Microbiol..

[27] Fernández-Fernández R., Elsherbini A.M.A., Lozano C., Martínez A., De Toro M., Zarazaga M., Peschel A., Krismer B., Torres C. (2023). Genomic Analysis of Bacteriocin-Producing Staphylococci: High Prevalence of Lanthipeptides and the Micrococcin P1 Biosynthetic Gene Clusters. Probiotics Antimicrob. Prot..

[28] Fernández-Fernández R., Lozano C., Fernández-Pérez R., Zarazaga M., Peschel A., Krismer B., Torres C. (2023). Detection of Micrococcin MP1 in commensal and environmental staphylococcal isolates with high antimicrobial activity against MRSA. Int. J. Antimicrob. Agents.

[29] Soltani S., Hammami R., Cotter P.D., Rebuffat S., Said L.B., Gaudreau H., Bédard F., Biron E., Drider D., Fliss I. (2021). Bacteriocins as a new generation of antimicrobials: Toxicity aspects and regulations. FEMS Microbiol. Rev..

[30] Al Atya A.K., Abriouel H., Kempf I., Jouy E., Auclair E., Vachée A., Drider D. (2016). Effects of Colistin and Bacteriocins Combinations on the In Vitro Growth of *Escherichia coli* Strains from Swine Origin. Probiotics Antimicrob. Prot..

[31] LeBel G., Piché F., Frenette M., Gottschalk M., Grenier D. (2013). Antimicrobial activity of nisin against the swine pathogen *Streptococcus suis* and its synergistic interaction with antibiotics. Peptides.

[32] Diez-Gonzalez F. (2007). Applications of bacteriocins in livestock. Curr. Issues Intest. Microbiol..

[33] Joerger R. (2003). Alternatives to antibiotics: Bacteriocins, antimicrobial peptides and bacteriophages. Poult. Sci..

[34] Vandeplas S., Dauphin R.D., Beckers Y., Thonart P., Théwis A. (2010). *Salmonella* in Chicken: Current and Developing Strategies to Reduce Contamination at Farm Level. J. Food Prot..

[35] Malinowski E., Krumrych W., Markiewicz H. (2019). The effect of low intensity laser irradiation of inflamed udders on the efficacy of antibiotic treatment of clinical mastitis in dairy cows. Vet. Ital..

[36] Fernández L., Delgado S., Herrero H., Maldonado A., Rodríguez J.M. (2008). The Bacteriocin Nisin, an Effective Agent for the Treatment of Staphylococcal Mastitis During Lactation. J. Hum. Lact..

[37] Godoy-Santos F., Pinto M.S., Barbosa A.A.T., Brito M.A.V.P., Mantovani H.C. (2019). Efficacy of a Ruminal Bacteriocin Against Pure and Mixed Cultures of Bovine Mastitis Pathogens. Indian J. Microbiol..

[38] Pieterse R., Todorov S.D. (2010). Bacteriocins: Exploring alternatives to antibiotics in mastitis treatment. Braz. J. Microbiol..

[39] Heilbronner S., Krismer B., Brötz-Oesterhelt H., Peschel A. (2021). The microbiome-shaping roles of bacteriocins. Nat. Rev. Microbiol..

[40] Bastos M.C., Ceotto H., Coelho M.L., Nascimento J.S. (2009). Staphylococcal antimicrobial peptides: Relevant properties and potential biotechnological applications. Curr. Pharm. Biotechnol..

[41] Wladyka B., Piejko M., Bzowska M., Pieta P., Krzysik M., Mazurek Ł., Guevara-Lora I., Bukowski M., Sabat A.J., Friedrich A.W. (2015). A peptide factor secreted by *Staphylococcus pseudintermedius* exhibits properties of both bacteriocins and virulence factors. Sci. Rep..

[42] Ovchinnikov K.V., Kranjec C., Telke A., Kjos M., Thorstensen T., Scherer S., Carlsen H., Diep D.B. (2021). A Strong Synergy Between the Thiopeptide Bacteriocin Micrococcin P1 and Rifampicin Against MRSA in a Murine Skin Infection Model. Front. Immunol..

[43] Degiacomi G., Personne Y., Mondésert G., Ge X., Mandava C.S., Hartkoorn R.C., Boldrin F., Goel P., Peisker K., Benjak A. (2016). Micrococcin P1—A bactericidal thiopeptide active against *Mycobacterium tuberculosis*. Tuberculosis.

[44] Bennett S., Ben Said L., Lacasse P., Malouin F., Fliss I. (2021). Susceptibility to Nisin, Bactofencin, Pediocin and Reuterin of Multidrug Resistant *Staphylococcus aureus*, *Streptococcus dysgalactiae* and *Streptococcus uberis* Causing Bovine Mastitis. Antibiotics.

[45] Bennett S., Fliss I., Ben Said L., Malouin F., Lacasse P. (2022). Efficacy of bacteriocin-based formula for reducing staphylococci, streptococci, and total bacterial counts on teat skin of dairy cows. J. Dairy Sci..

[46] Marques-Bastos S.L.S., Coelho M.L.V., De Sousa Santos I.N., Moreno D.S.A., Barrias E.S., De Mendonça J.F.M., Mendonça L.C., Lange C.C., De Paiva Brito M.A.V., Do Carmo De Freire Bastos M. (2023). Effects of the natural antimicrobial peptide aureocin A53 on cells of *Staphylococcus aureus* and *Streptococcus agalactiae* involved in bovine mastitis in the excised teat model. World J. Microbiol. Biotechnol..

[47] Wei M., Flowers L., Knight S.A.B., Zheng Q., Murga-Garrido S., Uberoi A., Pan J.T.C., Walsh J., Schroeder E., Chu E.W. (2023). Harnessing diversity and antagonism within the pig skin microbiota to identify novel mediators of colonization resistance to methicillin-resistant *Staphylococcus aureus*. mSphere.

[48] Eveno M., Savard P., Belguesmia Y., Bazibet L., Gancel F., Drider D., Fliss I. (2021). Compatibility, Cytotoxicity, and Gastrointestinal Tenacity of Bacteriocin-Producing Bacteria Selected for a Consortium Probiotic Formulation to Be Used in Livestock Feed. Probiotics Antimicro Prot..

[49] Reuben R.C., Roy P.C., Sarkar S.L., Alam R.U., Jahid I.K. (2019). Isolation, characterization, and assessment of lactic acid bacteria toward their selection as poultry probiotics. BMC Microbiol..

[50] García-Vela S., Ben Said L., Soltani S., Guerbaa R., Fernández-Fernández R., Ben Yahia H., Ben Slama K., Torres C., Fliss I. (2023). Targeting Enterococci with Antimicrobial Activity against *Clostridium perfringens* from Poultry. Antibiotics.

[51] Meyburgh C., Bragg R., Boucher C. (2017). *Lactococcus garvieae*: An emerging bacterial pathogen of fish. Dis. Aquat. Org..

[52] Chapman B., Gunter C. (2018). Local Food Systems Food Safety Concerns. Microbiol. Spectr..

[53] O’Sullivan L., Ross R.P., Hill C. (2002). Potential of bacteriocin-producing lactic acid bacteria for improvements in food safety and quality. Biochimie.

[54] Petruzzi L., Capozzi V., Berbegal C., Corbo M.R., Bevilacqua A., Spano G., Sinigaglia M. (2017). Microbial Resources and Enological Significance: Opportunities and Benefits. Front. Microbiol..

[55] Rojo-Bezares B., Sáenz Y., Zarazaga M., Torres C., Ruiz-Larrea F. (2007). Antimicrobial activity of nisin against *Oenococcus oeni* and other wine bacteria. Int. J. Food Microbiol..

[56] Fletcher J.T., Gaze R.H. (2008). Mushroom Pest and Disease Control: A Colour Handbook.

[57] Largeteau M.L., Savoie J.M. (2010). Microbially induced diseases of *Agaricus bisporus*: Biochemical mechanisms and impact on commercial mushroom production. Appl. Microbiol. Biotechnol..

[58] Gea F.J., Navarro M.J., Santos M., Diánez F., Carrasco J. (2021). Control of fungal diseases in mushroom crops while dealing with fungicide resistance: A review. Microorganisms.

[59] Vieira F.R., Pecchia J.A., Segato F., Polikarpov I. (2019). Exploring oyster mushroom (*Pleurotus ostreatus*) substrate preparation by varying phase I composting time: Changes in bacterial communities and physicochemical composition of biomass impacting mushroom yields. J. Appl. Microbiol..

[60] Carrasco J., García-Delgado C., Lavega R., Tello M.L., De Toro M., Barba-Vicente V., Rodríguez-Cruz M.S., Sánchez-Martín M.J., Pérez M., Preston G.M. (2020). Holistic assessment of the microbiome dynamics in the substrates used for commercial champignon (*Agaricus bisporus*) cultivation. Microb. Biotechnol..

[61] Just-Baringo X., Albericio F., Álvarez M. (2014). Thiopeptide Antibiotics: Retrospective and Recent Advances. Mar. Drugs.

[62] O’Sullivan J.N., Rea M.C., O’Connor P.M., Hill C., Ross R.P. (2019). Human skin microbiota is a rich source of bacteriocin-producing staphylococci that kill human pathogens. FEMS Microbiol. Ecol..

