# Supplementary material for: Bacteriocin-Producing Staphylococci and Mammaliicocci Strains for Agro-Food and Public Health Applications with Relevance of Micrococcin P1
Source: Antibiotics (Basel). 2025 Jan 16;14(1):97. doi: 10.3390/antibiotics14010097 (PMC11763047; doi:10.3390/antibiotics14010097)
Supplement: Supplementary file 1 [file antibiotics-14-00097-s001.zip › antibiotics-3369178-supplementary.pdf]

Supplementary Material

Table S1. Characteristics of the 15 bacteriocin-producing (BP) staphylococci and mammaliicocci (S/M) strains included in this study [a].

ID Code	Specie	Origin	Bacteriocin detected	Antibiotic Resistance		Virulence Content	Molecular Typing (*spa*-ST-CC/*agr*) [d]
				Phenotype [b]	Genotype		
C5802	*S. aureus*	Environmental-water	Micrococcin P1, Lactococcin972	PEN	*bla*Z	*luk*MF΄, *luk*ED, *etD*$_2$	t843-ST130-CC130/III
C5835	*S. hominis*	Environmental-water	Micrococcin P1	Susceptible	-	-	-
X3011	*M. sciuri*	Food-Chicken	Micrococcin P1, Lactococcin972	ERY-CLI-CIP-SXT	*sal*(A) [c], *dfr*A, *erm*(B)	-	-
X3041	*M. sciuri*	Food-Chicken	Micrococcin P1, Lactococcin972	CLI-FA	*sal*(A) [c], *erm*(B), *lnu*(A)	-	-
C8189	*S. pseudintermedius*	Human-Dog	BacSp222	ERY-CLI	*erm*(B)	*luk*S/F-I, *siet*	ST241/III
C8478	*S. pseudintermedius*	Human-Dog	BacSp222	ERY-CLI	*erm*(B)	*luk*S/F-I, *siet*	ST241/III
C8479	*S. pseudintermedius*	Human-Dog	BacSp222	ERY-CLI	*erm*(B)	*luk*S/F-I, *siet*	ST241/III
C9838	*S. chromogenes*	Wild animal-mammal	Circular	Susceptible	-	-	-
C8609	*S. aureus*	Wild animal-mammal	BSA, Lactococcin972	Susceptible	-	-	t11225-CC425/II
X3410	*S. aureus*	Food-Chicken	BacCH91	Susceptible	-	-	t10234/I
C6770	*S. aureus*	Wild animal-mammal	Lanthipeptide	Susceptible	-	-	t1125-CC5/II
X2969	*S. warneri*	Food-Chicken	Epilancin15X-Like	Susceptible	-	-	-
X3009	*S. epidermidis*	Food-Chicken	Lanthipeptide -New	ERY-FA	*msr*(A), *mph*(C)	-	ST1025
C9832	*S. simulans*	Wild animal-mammal	Lanthipeptide-New	TET-FA	*tet*(K)	-	-
C9585	*S. hyicus*	Wild animal-mammal	LanthipeptideV-like	Susceptible	-	-	-

[a]Data of the strains were obtained in previous studies [27,28].
[b]Antibiotics tested were the following ones: PEN: penicillin; cefoxitin; oxacillin; ERY: erythromycin; CLI: clindamycin; CIP: ciprofloxacin; TET: tetracycline; gentamicin, tobramycin; linezolid; chloramphenicol; SXT, trimethoprim-sulfamethoxazole; FA: fusidic acid; and mupirocin.
[c]*sal*(A): intrinsic resistance gene in *M. sciuri.*
[d]ST, sequence type; CC, clonal complex.

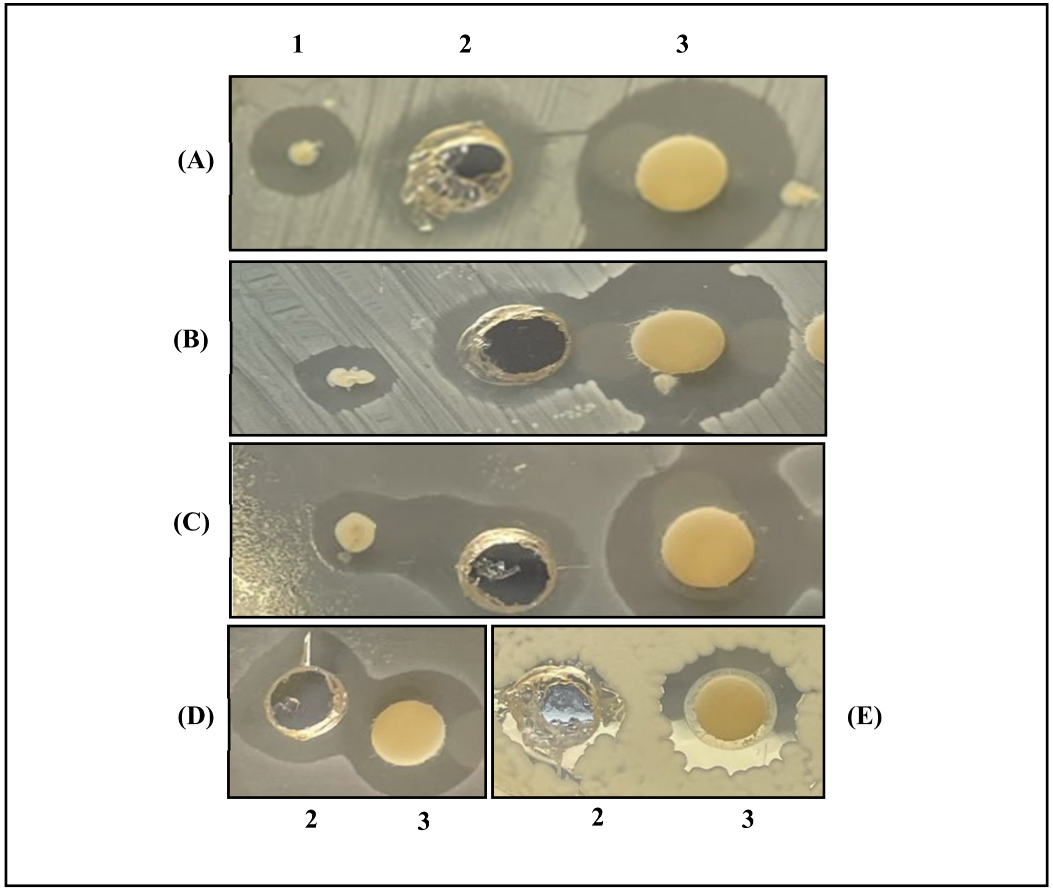

Figure S1. Antimicrobial activity of five bacteriocin-producing staphylococci and mammaliicocci strains against different indicator bacteria represented from left to right (1-3): 1.- bacteria (*spot-on-lawn*), 2. cell-free-supernatant (diffusion assay), and 3. butanol extracts. (A) *Staphylococcus aureus* C5802 against MRSA X3963; (B) *Staphylococcus hominis* C5835 against *Enterococcus faecalis* C9951; (C) *Mammaliicoccus sciuri* X3011 against *Listeria monocytogenes*; (D) *Staphylococcus aureus* C8609 against *Streptococcus gallolyticus*; (E) *Mammaliicoccus sciuri* X3041 against *Bacillus cereus.*

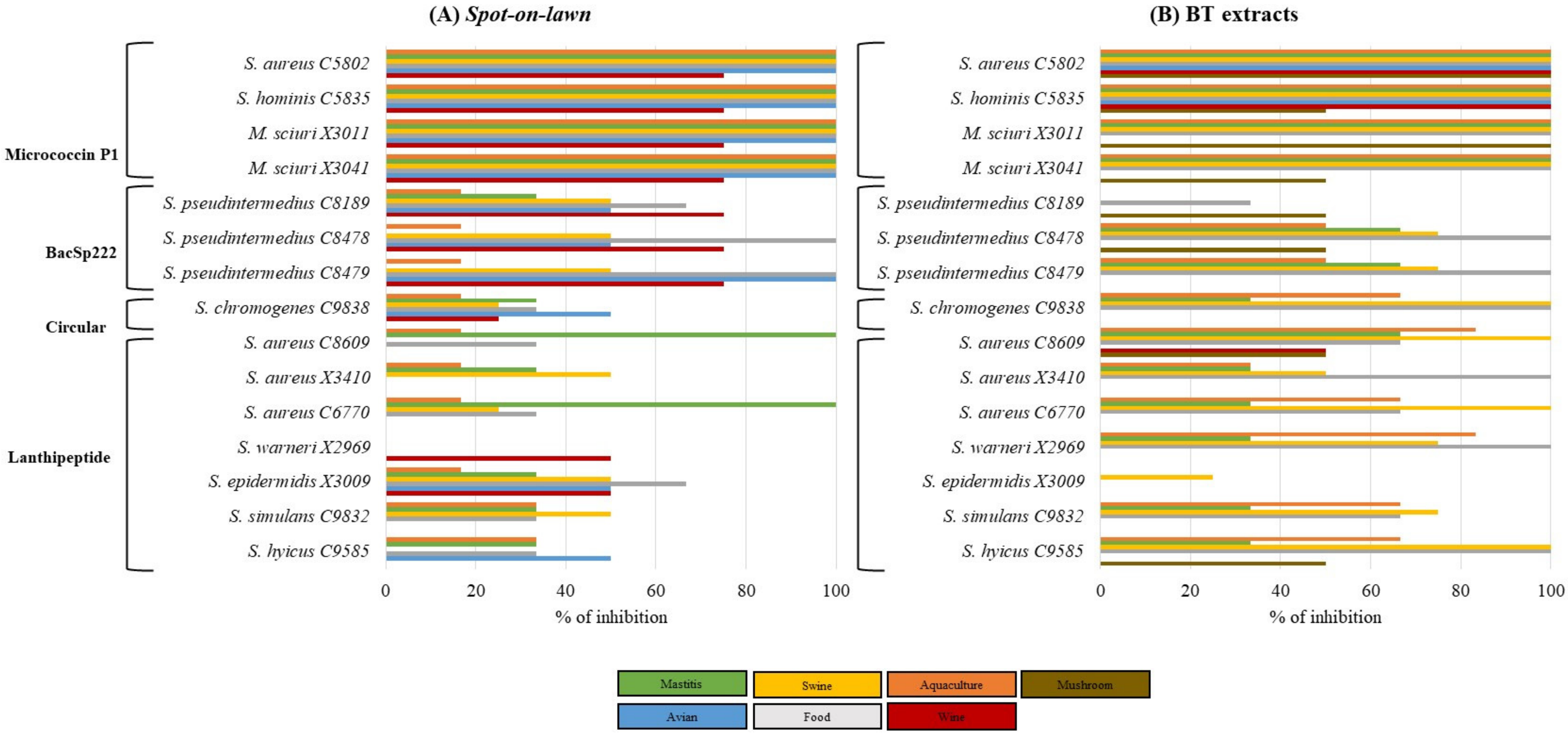

Figure S2: Spectrum of antimicrobial activity of the 15 bacteriocin-producing (BP) staphylococci/mammaliicocci (S/M) strains against the collection of microorganisms used as indicators considering the seven categories of application for which they were selected (marked in different colors). The 15 BP-S/M strains were grouped according to the type of bacteriocin produced. The figure represents the percentage of indicator strains inhibited by each of the BP staphylococci organized by the two methods (number of indicators tested): (A): *spot-on-lawn* (*n* = 22); (B): butanol extracts (BT, *n* = 24).
